# 

**Published:** 1898-10-22

**Authors:** 


**Notices of Births, Deaths, and Marriages** are inserted in this column at a charge of 2s. 6d. for an announcement not exceeding four lines.

### NOTICE TO CORRESPONDENTS.

All MS., Letters, Books for Review, and other matters intended for the Editor should be addressed The Editor, "The Hospital," The Hospital Building, 28 & 29, Southampton Street, Strand, W.C.

The Editor cannot undertake to return rejected MS., even when accompanied by stamped directed envelope.

### Notice to Advertisers and Subscribers.

All Advertisements, Orders for Copies of The Hospital, and Business Communications should be addressed to **THE MANAGER (Not to the Editor)**, The Hospital, The Hospital Building, 28 & 29, Southampton Street, Strand, London, W.C.

The Cost of Subscription, post free, is as follows:—Per week, 2½d.; per quarter, 2s. 8d.; per half-year, 5s. 3d.; per annum, 10s. 6d. Foreign:—Per annum, 15s. 2d., payable, in all cases, in advance.

**NOTICE.—Vol. XXIV. of "The Hospital" (April, 1898, to September, 1898), in handsome cloth binding, will shortly be on sale at the Office of this Journal, price 7s. 6d. Cases for binding the Numbers contained in this Volume 1s. 6d. Index to Vol. XXIV., 2½d., post free.**

**The Monthly Part for November will be ready November 1st. Price 6d., by post 8d.**

### SCRAPS AND GLEANINGS.

Three additional surgeons have been appointed to the staff of the Lowestoft Hospital.

A tender of £1,749 has been accepted for the erection of the Braintree Infectious Hospital.

The Badmain Cottage Hospital (New South Wales) received £120 this year from the Saturday Hospital Fund.

A local newspaper reports that the House Committee of the Swansea Hospital have passed a vote of censure upon the chief ophthalmic surgeon, who is stated to have refused the use of the eye department for the general out-patients, who cannot use their own quarters owing to structural alterations.

The two days' bazaar at Plymouth in aid of the funds of the South Devon and East Cornwall Hospital realised nearly £1,400.

Dr. H. E. Birch, of St. Thomas's and the London Hospitals, has been chosen to fill the post of assistant resident surgeon to the Buluwayo Hospital.

The half-yearly meeting of the governors of the Royal Orthopædic Hospital was held on September 28th. The report showed that the first half of 1898 had brought an increase in income over the same period of 1897, which increase was due to an improvement in donations and also to an award from a trust fund. Unfortunately, annual subscriptions showed a decrease.

Second Edition. Price 6d.,

**THE SCHOTT TREATMENT FOR CHRONIC HEART DISEASES.**

By RICHARD GREENE, F.R.C.P.Ed.

London: The Scientific Press, Ltd., 28 & 29, Southampton Street, Strand, W.C.

### WORKS FOR NURSES.

**A MANUAL FOR HOSPITAL NURSES** AND OTHERS ENGAGED IN ATTENDING ON THE SICK. By EDWARD J. DOMVILLE, L.R.C.P., M.R.C.S. Eighth Edition. Crown 8vo., 2s. 6d.

**A MANUAL OF NURSING, MEDICAL** AND SURGICAL. By C. J. CULLINGWORTH, M.D., F.R.C.P. Third Edition. Fcap. 8vo., 2s. 6d.

**A SHORT MANUAL FOR MONTHLY** NURSES. By C. J. CULLINGWORTH, M.D. Fourth Edition. Revised by M. A. ATKINSON. Fcap. 8vo., 1s. 6d.

**NOTES ON GYNÆCOLOGICAL NURSING.** By J. B. HELLIER, M.D. Fcap. 8vo., 1s. 6d.

**HINTS ON ELEMENTARY PHYSIOLOGY.** By FLORENCE A. HAIG-BROWN. With a Preface by Dr. ORD. With 21 Illustrations. Fcap. 8vo., 1s. 6d.

**LECTURES ON MEDICINE TO NURSES.** By HERBERT E. CUFF, M.D. Second Edition. With 29 Illustrations. Crown 8vo., 3s. 6d.

**ANTISEPTIC PRINCIPLES FOR NURSES.** By C. E. RICHMOND, F.R.C.S. Fcap. 8vo., 1s.

**A SHORT DICTIONARY OF MEDICAL** TERMS. 2s. 6d.

**CHAVASSE'S ADVICE TO A MOTHER** ON THE MANAGEMENT OF HER CHILDREN, and on the Treatment on the Moment of their more Pressing Illnesses and Accidents. Fifteenth Edition by Dr. GEORGE CARPENTER, Physician to the Evelina Hospital for Sick Children. 2s. 6d.

**CHAVASSE'S ADVICE TO A WIFE ON** THE MANAGEMENT OF HER OWN HEALTH, and on the Treatment of Some of the Complaints incidental to Pregnancy, Labour, and Suckling. With an Introductory Chapter especially addressed to a Young Wife. Fourteenth Edition, revised by Dr. FANCOURT BARNES, Consulting Physician to the British Lying-in Hospital. 2s. 6d.

London: J. and A. CHURCHILL,
7, Great Marlborough Street.

NEW EDITION.

### NURSES' REPORT BOOK,

WITH TEMPERATURE CHART, &c.

A most useful Case Book for Nurses. Arranged for Day and Night Reports for Three Weeks. Specially designed that each detail may be seen at a glance by the Medical Attendant.

Price **7d.** each, or **6s. 6d.** per doz., post free.

To be obtained only of

Miss LÖHR, Cottage Hospital, Potter's Bar.

